# Evaluation of the Graves' Orbitopathy-Specific Quality of Life Questionnaire in the Mainland Chinese Population

**DOI:** 10.1155/2019/7602419

**Published:** 2019-03-18

**Authors:** Peng Zeng, Shu-xian Fan, Zi-jing Li, Yuan-yu Peng, Yu-xin Hu, Ming-tong Xu, Mei Wang

**Affiliations:** ^1^Department of Ophthalmology, Guangdong Provincial Key Laboratory of Malignant Tumor Epigenetics and Gene Regulation, Sun Yat-sen Memorial Hospital, Sun Yat-sen University, Guangzhou 510120, China; ^2^Department of Endocrinology, Sun Yat-sen Memorial Hospital, Sun Yat-sen University, Guangzhou 510120, China

## Abstract

**Purpose:**

To study the clinical significance of the Graves' orbitopathy-specific quality of life (GO-QOL) questionnaire in mainland Chinese patients.

**Methods:**

A cross-sectional study was performed at the Ophthalmology Department of the Sun Yat-sen Memorial Hospital from April 2017 to April 2018. Eighty-eight consecutive Graves' orbitopathy (GO) patients completed the two subscales of the GO-QOL questionnaire: visual functioning and appearance. The disease severity of GO was measured by the European Group on Graves' Orbitopathy (EUGOGO) classification, and clinical activity was evaluated by the clinical activity score (CAS).

**Results:**

The mean scores of GO-QOL questionnaire for the visual functioning and appearance subscales were 68.4 ± 31.2 and 62.0 ± 27.4, respectively. Lower QOL scores for the visual functioning subscale were significantly correlated with disease severity, the CAS and diplopia (all *p* < 0.05). Lower QOL scores for appearance were significantly correlated with the CAS (*p* < 0.05). Although no correlation was found between the appearance subscale scores and disease severity (*p*=0.407), a downward trend in the appearance subscale scores as the severity of GO increased from mild to sight-threatening GO was found.

**Conclusion:**

A strong correlation between disease severity and clinical activity has been shown in the GO-QOL questionnaire, suggested by the EUGOGO. The GO-QOL questionnaire is a simple and effective appraisal instrument in the evaluation of health-related QOL in the mainland Chinese patients with GO.

## 1. Introduction

Graves' orbitopathy (GO) is an autoimmune inflammatory disease that potentially causes sight-loss, disfigures appearance, and results in an obvious loss of quality of life. [1–3] About 25–50% of Graves' disease patients show clinical eye signs and/or symptoms, and those people suffer from altered appearance and/or impaired vision [4, 5].

The Graves' orbitopathy-specific quality of life (GO-QOL) questionnaire was specifically created for GO patients by Terwee et al. and his colleagues. [6] The questionnaire has been proven to be a reliable tool to evaluate the QOL of GO patients in various countries and regions with different languages. [1, 7–9]. Furthermore, the GO-QOL was used as an indicator to assess the subjective changes associated with clinical treatment effects. [10–14] The objectives of this study were to evaluate the QOL of the mainland Chinese GO patients and to estimate the relationship between GO-QOL scores and the severity and activity of the disease.

## 2. Materials and Methods

This cross-sectional study included 88 patients with GO who were followed up at the Sun Yat-sen Memorial Hospital, Sun Yat-sen University, Guangzhou, China, from April 2017 to April 2018. GO was diagnosed in the Department of Endocrinology and Ophthalmology based on the presence of typical eye signs and symptoms in a patient with Graves' disease. All clinical observations and objective measurements were consistently performed by one ophthalmologist (Mei Wang). Data collected included patients' age, sex, signs, symptoms, duration of GO, clinical activity, and severity classification of GO according to the European Group on Graves' Orbitopathy (EUGOGO), history and duration of thyroid disease, history of smoking, and other systemic and eye diseases. Ophthalmological data included a measurement of best-corrected visual acuity, a slit lamp examination of the anterior eye segment, and a fundus examination with a 90D lens. Proptosis was measured with a Hertel exophthalmometer. This study was conducted in accordance with the tenets of the Declaration of Helsinki, the protocol of this study was approved by the Sun Yat-sen Memorial Hospital, Sun Yat-sen University Committee, and informed consent was obtained from all participants.

The English version of the GO-QOL questionnaire was downloaded from the EUGOGO website and then translated into Chinese. [8] The questionnaire was previously described by Terwee et al. and his colleagues [6] and was specifically projected for GO patients. The GO-QOL questionnaires were completed by all patients by themselves during outpatient clinic consultation, and explanations for questions were presented to patients by the ophthalmologist if needed. The scoring method of GO-QOL has been shown in previous reports. [8] In brief, the GO-QOL contained two subscales of GO quality of life: visual functioning and appearance. Each subscale contained 8 items, and each item was scored on a 3-point scale: 1-severely, 2-a little, and 3- not at all. The scores on the two subscales were counted from 0 to 100 using the following formula: (total scores-#)/(2 × #) × 100, where # showed the number of completed items. In all scores, 100 represented the best health state while 0 represented the worst health state. The responses were scored as missing if participants could not complete the questions for any reason.

The inflammatory activity of GO, including (1) spontaneous retrobulbar pain, (2) pain on attempted upward or downward gaze, (3) redness of the eyelids, (4) redness of the conjunctiva, (5) swelling of the caruncle or plica, (6) swelling of the eyelids, and (7) swelling of the conjunctiva (chemosis), was measured by the clinical activity score (CAS). [15] The CAS scores of 3–7 indicated activity.

EUGOGO classification [16] was used to assess the severity of GO, which was divided into mild, moderate-severe, and sight-threatening categories. Mild disease was defined as minor lid retraction (<2 mm), mild soft-tissue involvement, exophthalmos <3 mm above what is considered normal for the patient's race and sex, no or intermittent diplopia, and corneal exposure responsive to lubricants. Moderate-to-severe GO was defined as lid retraction ≥2 mm, moderate or severe soft-tissue involvement, or exophthalmos ≥3 mm above what is considered normal for the patient's sex and race, with diplopia (inconstant or constant). Sight-threatening GO was defined as dysthyroid optic neuropathy (DON) and/or severe corneal exposure (large corneal epithelial and/or stromal defects) or corneal breakdown (corneal descemetocele or keratohelcosis perforation).

## 3. Statistical Analysis

Data were collected and entered into a computerized statistical software package (SPSS 22.0) in a standard fashion. Continuous variables are expressed as the means ± the standard deviations, and categorical variables are presented as percentages. The floor and ceiling effects of the GO-QOL were measured in terms of the percentage of GO patients. In previous studies, “significant” floor and ceiling effects were defined as >15% of patients and “substantial” floor and ceiling effects were defined as >30% of patients. [8, 17] These definitions were used in our study. Associations between the QOL score and the EUGOGO classification and between the QOL score and the CAS were analyzed using the Wilcoxon rank-sum test or the Kruskal–Wallis rank-sum test. A two-sided *p*-value less than 0.05 was considered statistically significant.

## 4. Results

Demographic and clinical data for all patients are shown in Table 1. More than half of the patients were female (58 cases, 65.9%), and the mean age was 41.5 ± 15.6 years. The average durations of thyroid disease and GO were 35.8 ± 52.3 months and 16.1 ± 23.7 months, respectively. Nearly 40% of the patients experienced diplopia.

The percentages of responses to each item of the GO-QOL are summarized in Table 2. The scores of GO-QOL for the visual functioning and appearance subscales were 68.4 ± 31.2 and 62.0 ± 27.4, respectively.

### 4.1. Visual Functioning Subscale

**Figure 1:**
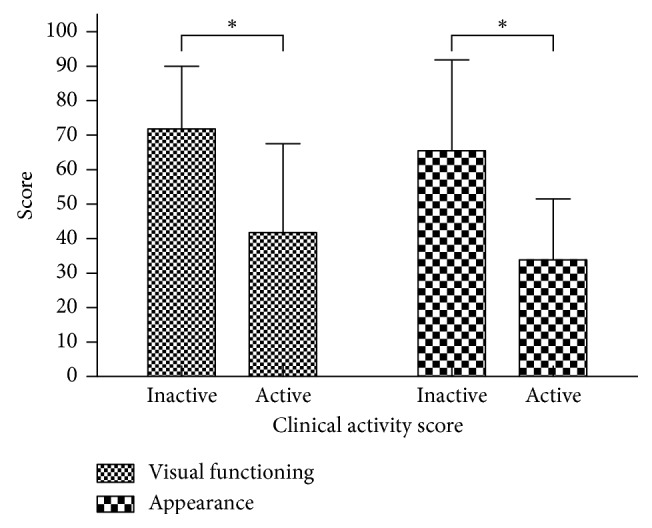
Clinical activity score on QOL score. The visual functioning scores of patients with active disease (41.9 ± 25.6) were obviously lower than those of patients who had inactive disease (71.8 ± 30.2, ^*∗*^*p*=0.003). The appearance subscale scores of patients with active disease (34.0 ± 17.5) were obviously lower than those of patients who had inactive disease (65.5 ± 26.3, ^*∗*^*p*=0.001).

**Figure 2:**
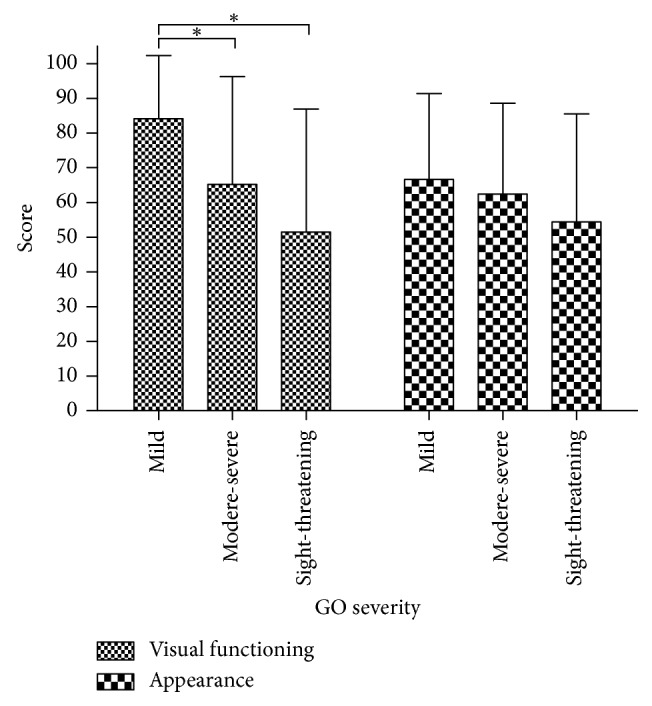
EUGOGO classification of the QOL score. The visual functioning subscale scores were markedly higher in patients with mild GO (84.1 ± 18.2) compared to those with moderate-severe (65.2 ± 31.1) and/or sight-threatening GO (51.6 ± 35.3): for mild versus moderate-severe GO, ^*∗*^*p*=0.006; for mild versus sight-threatening GO, ^*∗*^*p*=0.001; and for moderate-to-severe versus sight-threatening GO, *p*=0.225. The appearance subscale scores were markedly higher in patients with mild GO (66.6 ± 24.8) compared to those with moderate-severe (62.4 ± 26.2) and/or sight-threatening GO (54.5 ± 31.0). No correlation was found between appearance subscale scores and disease severity (^*∗*^*p*=0.407).

The most limited activities indicated in the visual functioning subscale were reading (58.0%), watching TV (58.0%), doing something you wanted to do (50.0%), and hobbies or pastimes (46.6%). In this study, the visual functioning subscale scores of GO-QOL were markedly related to clinical activity (*p*=0.003) and disease severity (*p*=0.002) (Figures 1 and 2). The visual functioning scores of patients with active disease (41.9 ± 25.6) were obviously lower than those of the patients who had inactive disease (71.8 ± 30.2), and the difference was significant (*p*=0.003). The visual functioning subscale scores were markedly higher in patients with mild GO (84.1 ± 18.2) compared to those with moderate-severe (65.2 ± 31.1) and/or sight-threatening GO (51.6 ± 35.3). The scores of GO-QOL visual functioning subscale were significantly different between mild versus moderate-severe GO (*p*=0.006) and mild versus sight-threatening GO (*p*=0.001). There were no differences between patients with moderate-to-severe versus sight-threatening GO (*p*=0.225). Additionally, a significant correlation was found between visual functioning scores and sex and diplopia (*p* < 0.001). However, there was no difference between visual functioning scores and age (*p*=0.214) or proptosis (*p*=0.546) in this study (Table 3).

### 4.2. Appearance Subscale

The most common psychosocial consequences of GO were “appearance has changed” (93.2%), “stared at in the streets” (62.5%), and “influence on self-confidence” (59.1%). The appearance subscale scores were markedly higher in patients with mild GO (66.6 ± 24.8) compared to patients with moderate-severe (62.4 ± 26.2) and/or sight-threatening GO (54.5 ± 31.0). The appearance subscale scores of GO-QOL were closely related to clinical activity (*p*=0.001) (Figure 1). This study showed there was a downward trend in the appearance subscale scores as GO severity increased from mild to sight-threatening GO; however, no correlation was found between appearance subscale scores and disease severity (*p*=0.407)(Figure 2). Additionally, there was no difference between appearance scores and sex (*p*=0.123), age (*p*=0.800), diplopia (*p*=0.696), or proptosis (*p*=0.261) in this study (Table 3).

## 5. Discussion

GO restricted patients' daily activities such as reading, watching television, and enjoying free time, as well as created a dysfunction in social roles and an impaired self-confidence because of the altered appearance. The GO-QOL survey was devised by Terwee et al. [6] and has been proven to accurately measure the QOL of GO-specific conditions. [1, 7–9] In this study, GO-QOL scores of mainland Chinese patients were strongly correlated with disease severity and clinical activity, especially in the visual functioning subscale. Although no significant correlation was found between the appearance subscale scores of GO-QOL and disease severity, there was a downward trend in the appearance subscale scores with the increasing severity of GO according to the EUGOGO classification. The results were similar to those reported in previous studies. [7, 8] Many signs and symptoms were used to assess the severity of GO. DON, corneal breakdown, and diplopia may seriously affect visual functioning. The degree of lid retraction, soft-tissue involvement, and proptosis may influence the appearance scores. In this study, diplopia was strongly correlated with visual functioning scores but not with appearance scores. This result was similar to that of previous studies. [7, 9] However, it was different from the Taiwanese (Chinese) study [8], which reported that the degree of diplopia was correlated only with appearance. A possible reason is that strabismus may alter appearance and affect self-confidence. The clinical activity score is frequently used as a clinical parameter of disease activity. When patients are in the active phase, the inflammatory symptoms and signs not only alter appearance but also decrease visual function, both of which make patients nervous and depressed, and the patients experience psychosocial problems.

The mean GO-QOL scores measured in this study were much higher than those measured in the Taiwanese (Chinese) survey (visual functioning, 58.4, and appearance, 54.5). The difference in QOL scores might be associated with different patient compositions.

Female patients attained higher visual functioning and lower appearance scores in this study compared with male patients. We speculate that physical attractiveness might be more important to women than it is to men among Chinese people. However, visual function might be more important to men than it is to women. Moreover, no relation was found between appearance score and age or visual functioning score and age. However, the results implied that patients aged 13–24 years attained lower appearance scores than older patients. One possible reason was that younger patients are vulnerable to the negative effects of GO on their emotions and appearance, which lead to a dysfunction in social roles and an impaired self-confidence.

Previous studies reported that significant ceiling effects were found on the two subscales and concluded that the GO-QOL lacked the sensitivity to distinguish an effect in mild GO patients. As we have known, a significant ceiling effect would limit the ability to measure improvements in the GO-QOL in GO patients. In this study, we found that no significant ceiling and floor effects were found on the two subscales of the GO-QOL. This result was similar to the Taiwanese study. [8] Many studies showed that the GO-QOL is used as an indicator to assess the improvement in GO patients after suitable treatment. [10, 13, 14] We deduced that the GO-QOL was an efficient instrument to evaluate patients with GO.

Our research has some limitations. First, this was a cross-sectional study, which makes it hard to assess dynamic changes in a patient's GO-QOL score over time. Second, GO severity and activity were evaluated by one ophthalmologist, which could improve reliability but might create systematic bias. Third, all participants were recruited from a single academic institution, and the backgrounds of participants in our clinic might be different from those of patients in a community setting.

In conclusion, GO could interfere with patient quality of life by damaging visual function and harming appearance. The GO-QOL survey is a simple and valuable tool to evaluate the severity and activity of GO in the mainland Chinese patients.

## Figures and Tables

**Table 1:** Demographic and clinical data for Grave's ophthalmopathy patients.

	*n*	%
Female/male	58/30	65.9/34.1
Age (y, mean ± SD, range)	41.5 ± 15.6 (13–73)	
Duration of autoimmune thyroid diseases (months, mean ± SD, range)	35.8 ± 52.3 (1–240)	
GO duration (months, mean ± SD, range)	16.1 ± 23.7 (1–120)	
Clinical activity score		
CAS < 3 (cases, %)	78	88.6
CAS ≥ 3 (cases, %)	10	11.4
Clinical severity		
Mild	30	34.1
Moderate-severe	37	42.1
Sight-threatening	21	23.9
Diplopia/no diplopia	36/52	40.9/59.1
Proptosis (mm, mean ± SD, range)	18.4 ± 2.9 (11–27)	

**Table 2:** Frequencies of responses to each item on the visual functioning and appearance subscales. (*n*=88).

Limitations in following activities	Seriously	Mildly	Not at all	Missing response
1. Bicycling	10.2	14.8	52.3	22.7^a^
2. Driving	12.5	10.2	28.4	48.9^a^
3. Moving around the house	8.0	17.0	75.0	0.0
4. Walking outdoors	11.4	23.9	64.7	0.0
5. Reading	21.6	36.4	42.0	0.0
6. Watching TV	23.9	34.1	42.0	0.0
7. Hobbies or pastimes	18.2	28.4	53.4	0.0
8. Hindered from doing something you wanted to do	19.3	30.7	50.0	0.0

Psychosocial results because of thyroid eye disease	Very much	A little	None	Missing response

1. Appearance has changed	52.3	40.9	6.8	0.0
2. Stared at in the streets	25.0	37.5	37.5	0.0
3. People react unpleasantly	5.7	26.1	68.2	0.0
4. Influence on self-confidence	26.1	33.0	40.9	0.0
5. Socially isolated	9.1	21.6	69.3	0.0
6. Influence on making friends	12.5	30.7	56.8	0.0
7. Appear less often in photos than before	28.4	29.6	42.0	0.0
8. Mask changes in your appearance	20.5	30.7	48.8	0.0

Data are provided as %. a: “never learned to ride a bike” and “no driver's license” were scored as missing.

**Table 3:** Results of the univariate analysis of Grave's ophthalmopathy patients.

Covariate	Visual functioning	*p*	Appearance	*p*
Sex		0.015		0.123
Female	73.5 ± 30.9		58.3 ± 28.7	
Male	58.4 ± 29.3		68.9 ± 24.6	

Age		0.214		0.800
13–24 y	77.5 ± 21.7		57.9 ± 27.1	
25–54 y	70.4 ± 29.7		63.3 ± 25.6	
55–73 y	56.2 ± 36.9		61.3 ± 31.4	

Diplopia		<0.001		0.696
No diplopia	81.2 ± 22.7		63.7 ± 24.8	
Diplopia	50.8 ± 32.7		59.5 ± 30.4	

Proptosis		0.546		0.261
≤20 mm	68.2 ± 29.9		63.6 ± 28.1	
>20 mm	68.9 ± 34.5		57.3 ± 24.4	

Clinical activity		0.003		0.001
Inactive	71.8 ± 30.2		65.5 ± 26.3	
Active	41.9 ± 25.6		34.0 ± 17.5	

Severity		0.002^a^		0.407
Mild	84.1 ± 18.2		66.6 ± 24.8	
Moderate-severe	65.2 ± 31.1		62.4 ± 26.2	
Sight-threatening	51.6 ± 35.3		54.5 ± 31.0	

^*∗*^Wilcoxon rank-sum test or Kruskal–Wallis rank-sum test. ^a^Severity: mild versus moderate-to-severe, *p*=0.006; mild versus sight-threatening, *p*=0.001; and moderate-to-severe versus sight-threatening, *p*=0.225 (least significant difference adjusted: *α* = 0.05/3 = 0.0167).

## Data Availability

The data used to support the findings of this study are included within the article.
